# RasGrf1: genomic imprinting, VSELs, and aging

**DOI:** 10.18632/aging.100354

**Published:** 2011-07-11

**Authors:** Mariusz Z. Ratajczak, Magda Kucia, Rui Liu, Dong-Myung Shin, Ewa Bryndza, Michal M. Masternak, Maciej Tarnowski, Janina Ratajczak, Andrzej Bartke

**Affiliations:** ^1^ Stem Cell Institute at James Graham Brown Cancer Center, University of Louisville, Louisville, KY 40202, USA; ^2^ Department of Physiology, Pomeranian Medical University, Szczecin, Poland and; ^3^ Department of Physiology, Southern Illinois University School of Medicine, Springfield, IL, USA

**Keywords:** Aging, longevity, IGF-1, RasGrf1, VSEL

## Abstract

Increase in life span in RasGrf1-deficient mice revealed that RasGrf1 deficiency promotes longevity. Interestingly, RasGrf1 is one of parentally imprinted genes transcribed from paternally-derived chromosome. Erasure of its imprinting results in RasGrf1 downregulation and has been demonstrated in a population of pluripotent adult tissues-derived very small embryonic like stem cells (VSELs), stem cells involved in tissue organ rejuvenation. Furthermore, based on recent observation that RasGrf1 signaling molecule is located downstream from insulin (Ins) and insulin like growth factor-1 (Igf-1) receptors, the extended life-span of RasGrf1^−/−^ mice may support beneficial effect of reduced Ins/Igf-1 signaling on longevity. Similarly, downregulation of RasGrf1 in VSELs renders them resistant to chronic Ins/Igf-1 signaling and protects from premature depletion from adult tissues. Thus, the studies in RasGrf1^−/−^ mice indicate that some of the imprinted genes may play a role in ontogenetic longevity and suggest that there are sex differences in life span that originate at the genome level. All this *in toto* supports a concept that the sperm genome may have a detrimental effect on longevity in mammals. We will discuss a role of RasGrf1 on life span in context of genomic imprinting and VSELs.

## RasGrf1 in longevity studies

In the March issue of *Aging,* Borras et al. reported that RasGrf1 deficiency delays aging in mice, with the lifespan of RasGrf1^−/−^ animals increased by ~20% [1]. RasGrf1 is a small GTP exchange factor (GEF) for H-Ras encoded by the paternally imprinted gene located on mouse chromosome 9 and associated with post-natal growth [2]. RasGrf1 is expressed in mouse in the pancreatic islets and in some regions of the brain (e.g., hypothalalmus) [2], which explains its role in Ras activation in regulation of the pancreatic mass of insulin-producing β-cells, insulin secretion, and glucose homeostasis, as well as its involvement in some neuronal functions (e.g., an indirect effect on growth hormone synthesis and learning and memory). In addition to binding H-Ras, RasGrf1 shows other pleiotropic functions after binding to members of the R-Ras and RAC subfamilies [2].

Several possibilities should be considered in explaining how RasGrf1 deficiency prolongs lifespan in mice. Specifically, RasGrf1-deficient mice display lower levels of ROS and a reduction in the parameters of oxidative stress (i.e., glutathione, oxidized protein, and malondialdehyde levels), higher levels of sirtuin, and, what is very important, lower levels of circulating growth hormone (GH) and insulin/insulin like growth factor-1 (IGF-1) in peripheral blood [1]. Thus, in mice, RasGrf1 deficiency mimics the caloric restriction (CR) pathway, which is well known to have beneficial effects in extending lifespan. Evidence is accumulating, based on murine models (e.g., Laron dwarf mutants and normal mice under CR), for an association between low-IGF-1 signaling and extended longevity [3-4]. Conversely, as reported for GH-overexpressing transgenic mice that display high levels of circulating IGF-1, increased IGF-1 signaling accelerates the aging process [5-6].

Overall, the report by Borras *et al*. nicely corroborates Kono et. al, who reported that bimaternal mice (“sperm DNA free-derived mice”) exhibit a life span extended by ~30% [7]. These bimaternal mice were artificially produced with two sets of female genomes by modulating expression of two paternally imprinted loci: insulin-like growth factor-2 (IGF-2)-H19 and Dlk1-Gtl2, located on chromosomes 7 and 12, respectively. In these mice, however, RasGrf1 was repressed and this condition, as postulated for bimaternal mice [7] and confirmed now in RasGrf1^−/−^ mice [1], is presumably a cause of their extended lifespan. Moreover, bimaternal animals, like RasGrf1^−/−^ animals, exhibit smaller body size, a trait also observed in several other long-lived mouse models (e.g., Laron, Ames, and Snell dwarfs) [3]. Based on these reports, RasGrf1 has been identified as a gene that may regulate life span in mice. In further support of this notion, it is well known that mutations of CDC25, a RasGrf1 homolog in yeast [8] or mutations of the Ras pathways in *C. elegans* [9] also lead to extended life span. Finally, the RasGrf1 gene has recently been found to be associated with exceptional longevity in humans [10]. In sum, all these data have important, and not always fully appreciated, implications for parentally imprinted genes, including RasGrf1 in mice, that may regulate lifespan in mammals.

## Parentally imprinted genes and longevity

Genomic imprinting is an epigenetic program that results in expression of genes from only one of the two parental chromosomes [11]. Imprinted genes play a crucial role in embryogenesis, fetal growth, the totipotential state of the zygote, and the pluripotency of developmentally early stem cells. Overall, there are ~80 imprinted genes in mice (expressed from maternal or paternal chromosomes only) that play a important role in embryonic development. Based on the generation of the sperm DNA-free bimaternal mice mentioned above, a proper imprinting state of Igf-2-H19 and Dlk1-Gtl2 loci seems to be crucial for initiating proper embryogenesis.

The expression of imprinted genes is regulated by DNA methylation (imprinting) on differentially methylated (imprinted) regions (DMRs), which are CpG-rich cis-elements. While almost all imprinted genes are maternally imprinted, there are three known paternally imprinted genes in mice, which are Igf-2-H19, Dlk1-Gtl2, and RasGrf1. While paternally imprinted genes methylated on the haploid set of sperm chromosomes (Igf-2-H19, Dlk1-Gtl2, and RasGrf1) increase body mass and shorten life span, the same genes in a non-imprinted state on the haploid set of maternal chromosomes in oocytes contribute to smaller body size and extended life span.

The proper imprinting of paternal and maternal genes, which are combined in the zygote at the time of fertilization, is required for balanced expression (“dosage”) and to initiate embryogenesis. During development, all diploid somatic cells have a proper imprint imposed on chromosomes inherited from both of the parents, except precursors of gametes (primordial germ cells) that erase imprinting during their developmental migration to the genital ridges [12]. The erasure of imprinting makes these cells quiescent and prevents them from undergoing teratoma formation. Imprinting is reestablished in the germ line after the first meiotic division in precursor cells for sperm and oocytes when the chromosomes have been reduced to haploid number. These epigenetic changes occur in developing gonads, indicating the influence of sex hormones, which somehow regulate imprinting and expression of imprinted genes.

It is known that erasure of genomic imprint keeps pluripotent stem cells (e.g., primordial germ cells) from proliferation and teratoma formation [12]. Differences in imprinted genes may also explain why females live longer than males in many mammalian species, including humans. However, the reason why women have an advantage over men with regard to lifespan is still not fully clear. Future studies on sex-related epigenetic memory in somatic cells and changes in sex-dependent expression of imprinted genes may provide some clues to this phenomenon. On the other hand, it is also well known that mice cloned from somatic cells (e.g., by reproductive cloning) have a significantly shorter lifespan than mice derived from natural mating or derived as bimaternal mice by fusion of two female gametes.

The differentially methylated/imprinted DMR for the RasGrf1 gene is located upstream of its promoter, and is imprinted/methylated on paternal chromosomes and non-imprinted/non-methylated on maternal chromosomes. Methylation of this DMR prevents binding of the insulator protein CTCF to this region. If non-methylated, this DMR binds CTCF, which suppresses expression of RasGrf1. Therefore, RasGrf1 is transcribed from the paternal chromosome and is silent on the chromosome from the mother. This leads to the balanced "dosage" of RasGrf1 expression in diploid somatic cells from one of the 9^th^ murine chromosomes. Thus, parentally imprinted genes (Igf-2-H19, Dlk1-Gtl2, and RasGrf1) seem to be master regulators of development, and evidence is accumulating that they have also an impact on longevity.

## The paternally imprinted RasGrf1 gene regulates the number of VSELs

Our interest in RasGrf1 began a few years ago when we identified a population of dormant pluripotent stem cells in adult murine tissues, including bone marrow (BM), that we named very small embryonic-like stem cells (VSELs). Murine BM-derived VSELs: i) are very rare (~0.01% of nucleated BM cells); ii) are small in size (~3–5 μm); iii) express several pluripotent stem cell (PSC) markers, such as Oct4, Nanog, Rex-1, and SSEA-1; iv) possess large nuclei containing unorganized chromatin (euchromatin); and v) are capable of differentiation into cells from all three germ lineages *in vitro*. Furthermore, VSELs exhibit a significantly higher nuclear/cytoplasm (N/C) ratio and a lower cytoplasmic area compared to HSCs. We have already confirmed the true expression of Oct4 and Nanog in BM-derived VSELs by demonstrating an unmethylated, transcriptionally active open chromatin structure for both the Oct4 and Nanog promoters [13] in these cells.

We have postulated that these cells are a backup population of PSCs that supplies stem cells committed to particular tissues and thus plays a role in regeneration and rejuvenation of adult tissues. As we have demonstrated in BM, VSELs are precursor cells for hematopoietic stem cells (HSCs).

**Figure 1:**
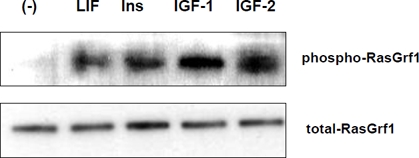
RasGrf1 phosporylation in murine embryonic ES-D3 cells. **Panel A**. RasGrf1 protein phophorylation at Ser929 was detected by western blot after stimulation for 5 min with LIF (50 ng/mL), insulin (10 ng/mL), IGF-1 (100 ng/mL), and IGF-2 (100 ng/ml). **Panel B**. The total level of Ras-Grf1 protein. The experiment was repeated twice with similar results.

**Figure 2:**
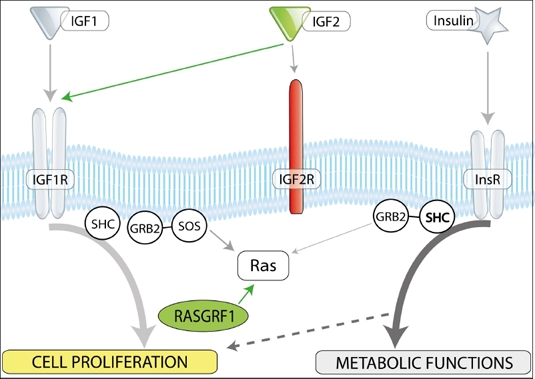
Insulin/IGF-1 and -2 signaling and imprinted genes. In mammals, there are three insulin factors (insulin, IGF-1, and IGF-2) that bind to two tyrosine kinase receptors, insulin receptor (InsR), and IGF-1 receptor (IGF-1R). Igf2R is a non-signaling mannose-type sink receptor for IGF-2. Depending on cell type, activation of InsR and IGF-1R lead to metabolic and proliferative responses. RasGrf1 is a small GTP exchange factor (GEF) and is involved in signaling from InsR and IGF-1R. VSELs, due to changes in the epigenetic state of parentally imprinted genes, show a decrease in Igf-2 and RasGrf1 expression (green) and Igf2R overexpression (red). These epigenetic changes in genes regulating insulin/Igf signaling keep VSELs quiescent in adult tissues. We hypothesize that chronic exposure to Ins/Igf accelerates premature depletion of VSELs, while RasGrf1 deficiency has the opposite effect.

We have recently identified a crucial genomic imprinting-related epigenetic mechanism that governs the VSEL quiescent state, preventing their proliferation and the spontaneous growth of teratomas [13]. This mechanism is based on the epigenetic changes in selected somatic-imprinted genes (*i.e.*, Igf-2-H19, RasGRF1, and IGF-2R) that are involved in insulin (Ins) and insulin-like growth factor signaling (IGF-1 and IGF-2). By erasing paternal imprints, VSELs downregulate expression of IGF-2, which seems to be an autocrine growth factor for these cells. Additionally, Ins/Igf signaling in VSELs is impaired due to erasure of the paternal imprinting of the RasGrf1 gene, which represses its expression. In support of this notion, we observed that in embryonic stem cells, RasGrf1 is indeed the downstream signaling protein for activated insulin and insulin growth factor-1 receptors (Figure 1). Furthermore, in addition to perturbed imprinting of paternally imprinted genes in VSELs, some of the maternally imprinted genes become, by contrast, hypermethylated. One of these genes is the IGF-2 receptor (IGF-2R), which serves as a molecular sink for IGF-2 and prevents its interaction with IGF-1 receptor (IGF-1R) [12]. The involvement of these paternally imprinted (Igf-2 and RasGrf1) and maternally imprinted (IGF-2R) genes in insulin and insulin-like growth factor signaling is depicted schematically in Figure 2.

Thus, impaired signaling from IGF-1R and InsR is responsible for keeping VSELs quiescent in adult tissues. It is well known that changes in Ins/Igf signaling have important implications for aging. As mentioned above i) IGF-1 signaling negatively regulates lifespan in worms, flies, and mammals and ii) IGF-1 and insulin level in blood is positively regulated by caloric uptake [4]. We hypothesize that chronic stimulation over time with insulin and IGF-1 leads to accelerated depletion of these cells in tissues [12]. We have demonstrated that the number of VSELs decreases with age in mice and correlates with their senescence [12], which could explain the aging process at the level of the PSCs residing in adult tissues (VSELs) [12]. A decrease in the number of these cells would directly impact the regenerative capacity of their progeny, the pool of tissue-committed stem cells that are more restricted in their ability to differentiate.

**Figure 3:**
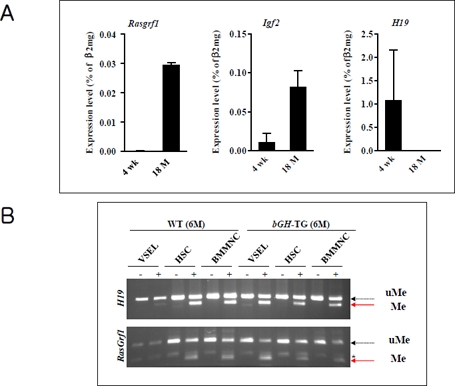
The change of genomic imprinting during VSEL ageing **Panel A**. RQ-PCR analysis of RasGrf1, Igf2, and H19 expression in VSELs isolated from 4-week-old and 18-month-old C57BL/6 mice. The relative expression level was represented as the percentage of β2 microglobin (β2mg) expression and shown as the mean ± SD. Please note reciprocal expression in Igf-2 (that promotes proliferation) and H19 (that encodes for mRNA giving rise to several miRNA negatively affecting cell proliferation). Both of these genes (Igf2-H19) are regulated in tandem by a common DMR that affects their expression - when one is silenced another one is upregulated. **Panel B**. Combined bisulfate-restriction analysis (COBRA) assay of Igf2-H19 DMR1 (upper panel) and RasGrf1 DMR (lower panel) by BstUI restriction enzyme in 6-month-old control wild type (WT) and 6-month-old transgenic bovine GH-overexpressing mice (bGH-TG). The COBRA assay was performed on VSELs, hematopoietic stem cells (HSC), and bone marrow mononuclear cells (BMMNC). “-” indicates bisulfate modification was not performed on the DNA (control); “+” indicates that DNA was subjected to bisulfate modification. The unmethylated DNA (uMe) was not cleaved, in contrast to methylated DNA (Me), because of a sequence change at the site recognized by a restriction enzyme after bisulfite reaction.

In fact, our studies performed on normal young (4-week-old) and old (2-year-old) mice revealed that the number of VSELs and their pluripotency decreases during ageing [14]. Accordingly, VSELs from young mice show erasure of DMRs for Igf-2-H19 and RasGrf1 loci and thus do not express IGF-2 and RasGrf1 (Figure 3 panel A). In contrast, VSELs from old mice show the somatic type of methylation at both Igf2-H19 and RasGRF1 loci, which increases expression of Igf-2 and RasGrf1 and, thus, their sensitivity to insulin factor signaling (Figure 3 panel A). This suggests that chronic Ins/Igf signaling via RasGrf1 may contribute to age-related depletion of VSELs and the senescence process. This in parallel corresponds with changes that lower expression of the pluripotency master-regulators, such as Oct4, Nanog, Sox2, Klf4, and cMyc. At the molecular level, the Oct4 promoter in VSELs as we have demonstrated becomes gradually hypermethylated with age and shows a closed chromatin structure [14].

Further evidence for the role of VSELs and imprinted genes on lifespan comes from our recent studies on murine models of longevity [15]. We observed that the number of VSELs in BM positively correlates with extended lifespan and both parameters are negatively affected by plasma IGF-1 level. In support of this observation, while long-living Laron dwarf mice with low circulating plasma IGF-1 levels have a significantly higher number of VSELs at age 2 years compared to their wild type (wt) littermates [14], short-living bovine GH-overexpressing transgenic mice with high levels of circulating IGF-1 display a reduced number of VSELs at age 6 months [15]. These changes in the number of VSELs were correlated with hypomethylation of DMRs at the Igf-2-H19 and RasGrf1 loci in Laron dwarfs and hypermethylation of these loci in GH-transgenic animals ([14, 15] and Figure 3 panel B). The corresponding reduction in VSEL number and increased hypermethylation of DMRs for Igf-2-H19 and RasGrf1 loci and expression of these genes was recently confirmed in wt mice that were injected for 2 months with GH or IGF-1 (submitted for publication).

Based on these data, we proposed that epiblast-derived VSELs, which are deposited in adult tissues as a population of most-primitive stem cells involved in tissue/organ rejuvenation, are depleted by chronic Ins/Igf signaling. Prolonged signaling by insulin, IGF-1, and IGF-2 (*e.g.*, due to high caloric uptake or as a result of GH overexpression in bGH transgenic mice) may lead to premature depletion of these cells in tissues. BM-residing VSELs are a backup population for HSCs and a decrease in the number of VSELs in BM will directly affect the pool of these cells. Further studies are required to see whether similar scenarios occur in other cases of tissue-committed stem cells (e.g., mesenchymal stem cells or satellite stem cells). A reverse phenomenon occurs in attenuated insulin, IGF-1, and IGF-2 signaling (e.g., due to caloric restriction, GHR mutations, or RasGrf1^−/−^ mice) where VSELs are protected from uncontrolled premature depletion. We hypothesize that while the activation of VSELs occurs via very finely tuned and controlled signals (e.g., bone morphogenic protein levels), uncontrolled and prolonged Ins/Igf signaling depletes these cells and, more importantly, may put them in danger of becoming cancer stem cell precursors. In fact, animals with enhanced Ins/Igf signaling have a much higher risk of tumor formation [5] compared, for example, to Laron dwarf mice or Laron dwarf patients, which have very low levels of circulating IGF-1 and are highly resistant to development of neoplastic diseases [3, 16].

## CONCLUSIONS

In conclusion, longevity studies in RasGrf1-deficient mice have demonstrated indirectly that imprinted genes play a crucial role in ontogenetic longevity. These studies also suggest that there are sex differences in longevity that originate at the genome level, implying that the sperm genome has a detrimental effect on longevity in mammals. It will be interesting to see whether RasGrf1-overexpressing mice display a shortened lifespan and whether small molecule inhibitors of Ras signaling will have beneficial effects on longevity in mice and perhaps humans as well. It would also be interesting to measure directly the number of VSELs and the state of parentally imprinted genes both in RasGrf1^−/−^ and RasGrf1-overexpressing mice. In sum, longevity studies have increased in sophistication to the point where explanations can now be sought at the level of imprinted genes, which are the master regulators of development, and their epigenetic regulation.
